# Prepartum nutrient intake and colostrum yield and composition in ruminants

**DOI:** 10.1093/af/vfad031

**Published:** 2023-06-12

**Authors:** Koryn S Hare, Amanda J Fischer-Tlustos, Katharine M Wood, John P Cant, Michael A Steele

**Affiliations:** Department of Animal Biosciences, Animal Science and Nutrition, Ontario Agricultural College, University of Guelph, Guelph, ON, CanadaN1G 1Y2; Department of Animal Biosciences, Animal Science and Nutrition, Ontario Agricultural College, University of Guelph, Guelph, ON, CanadaN1G 1Y2; Department of Animal Biosciences, Animal Science and Nutrition, Ontario Agricultural College, University of Guelph, Guelph, ON, CanadaN1G 1Y2; Department of Animal Biosciences, Animal Science and Nutrition, Ontario Agricultural College, University of Guelph, Guelph, ON, CanadaN1G 1Y2; Department of Animal Biosciences, Animal Science and Nutrition, Ontario Agricultural College, University of Guelph, Guelph, ON, CanadaN1G 1Y2

**Keywords:** cattle, colostrum, immunoglobulin G, prepartum, nutrition, sheep

**Implications**

- Many cattle and sheep do not produce enough colostrum for their offspring.
- Prepartum nutrient intake is an accessible strategy for producers to influence colostrum production.
- Greater prepartum starch intake can influence colostrum composition and increase colostrum yield for beef cattle and ewes.
- Colostrogenesis is sensitive to fat intake, dependent on the dietary fatty acid composition: greater linoleic acid intake often increases colostrum antibody concentration.
- Colostral bioactive compounds are frequently altered by a prepartum diet without changes in overall colostrum composition. Prepartum nutrient intake could be strategically used to maximize beneficial compounds for the newborn.

## Introduction

Adequate colostrum production is essential to ensure that neonates consume appropriate quantities of immunoglobulin G (**IgG**), macronutrients, and bioactive compounds that promote physiological maturation and support the immune system (Fischer-Tlustos et al., 2021a). Dairy cattle produce relatively large colostrum yields at calving (mean: 4.6–7.9 kg; Cabral et al., 2016) compared to beef cattle (mean: 2.64 kg; McGee and Earley, 2018; Hare et al., 2021) and ewes (range: approximately 150–650 g; Banchero et al., 2015). However, variation in colostrum yield for dairy cattle is substantially higher and demonstrates a left-skewed distribution (Cabral et al., 2016), meaning that inadequate colostrum production (<6 kg, based on the mass of colostrum needed to feed a calf two meals; Westhoff et al., 2023) is more common than excessive production. In Westhoff et al. (2022), 73.4% of primiparous and 61.5% of multiparous cattle produced inadequate colostrum volumes. Beef and sheep producers do not typically quantify colostrum yield due to differences in production systems and it is unlikely that mass output would be limiting for the transfer of passive immunity in these species based on their elevated colostrum IgG concentrations (Swanson et al., 2008; Hare et al., 2021). That said, inadequate colostrum production is a possibility for high-fecundity ewes (Banchero et al., 2015) and, furthermore, the metabolizable nutrient output becomes relatively more important for beef calves and lambs to support metabolism, especially in challenging climates.

There has been substantial interest in uncovering which maternal, managerial, and environmental factors affect colostrum yield and IgG concentration in ruminants. Plenty of research has been conducted with dairy cattle and notable factors associated with colostrum yield and IgG concentration include parity (Soufleri et al., 2021), dry period length, maximum temperature humidity index and photoperiod, calf sex, previous 305-d milk yield (Westhoff et al., 2022), genetic heritability (Soufleri et al., 2021), blood analytes (Immler et al., 2021; Rossi et al., 2023), and prepartum nutrient intake (Mann et al., 2016). Many of these factors either cannot be adjusted for or are challenging to adjust through prepartum management (i.e., parity, environment, calf sex, or 305-d milk yield), or have an associated time-lag (genetic selection) that hinders their initial utility for producers. However, prepartum nutrient intake is an accessible target for dairy, beef, and sheep producers to affect and, potentially, increase colostrum production and IgG yield.

Most research evaluating how prepartum nutrient intake affects colostrum production has mainly focused on global nutrient under- or over-provision prior to parturition. Imposing mid to late-gestation nutrient restriction reduced first-milking colostrum yield (within 1 h postcalving) in beef cattle (Logan, 1977; Petrie et al., 1984) without altering colostrum IgG concentration (Logan, 1977; Petrie et al., 1984) but compromised total IgG yield (Petrie et al., 1984). Similarly, severe gestational nutrient restriction (60% requirements) in ewes proportionally reduced first-milking colostrum yield (within 1 h postlambing) while increasing IgG concentration and suppressing IgG yield (Swanson et al., 2008). Interestingly, providing nutrients in excess of requirements by 40% also reduced colostrum yield by a comparable magnitude, without altering colostral IgG concentration, but consequently lessening IgG mass output (Swanson et al., 2008). It is clear that prepartum nutrient intake can impact the quantity of colostrum produced by ruminants, as well as the IgG concentration and total IgG yield. Yet, when total feed intake is manipulated, the causative effect cannot be discerned. That is, one cannot differentiate whether these responses are due to metabolizable energy (**ME**) or protein (**MP**) intake, or dietary components such as starch, neutral detergent fiber (**NDF**), or fat.

McGee and Earley (2018) reviewed factors that influence colostrum yield and IgG concentration in beef cattle, including nutrient intake, without differentiating between dietary components. Similarly, Banchero et al. (2015) summarized compelling data underlining the importance of starch intake with late-gestation ewes, though relatively less consideration was given to alternate dietary components. To our knowledge, similar reviews have not been published for dairy cattle. Therefore, the aim of this review is to summarize how dairy and beef cattle and ewes respond to prepartum carbohydrate, protein, and fat intake. We discuss responses in terms of colostrum yield, composition (fat, protein, lactose, and IgG concentrations and bioactive components), and total component yields.

### Prepartum dietary carbohydrate intake

**Figure 1.**
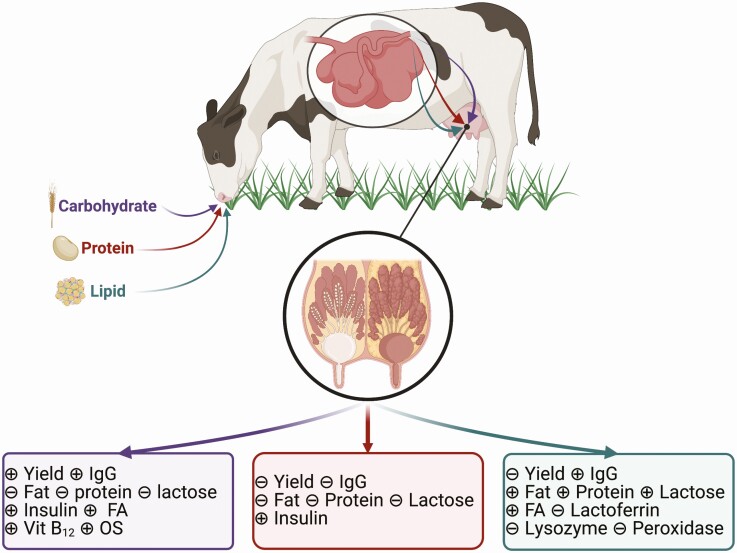
A schematic representation of how dietary carbohydrate (color-coded purple), protein (color-coded maroon), and fat (color-coded teal) intake affects colostrum yield and composition (immunoglobulin G [**IgG**], macronutrient, and bioactive component concentrations) produced by dairy and beef cattle. ⊝ Indicates that the dietary component does not affect that colostrum parameter whereas ⊕ indicates that the colostrum parameter is affected by that dietary component. The figure was created with Biorender.com

**Figure 2.**
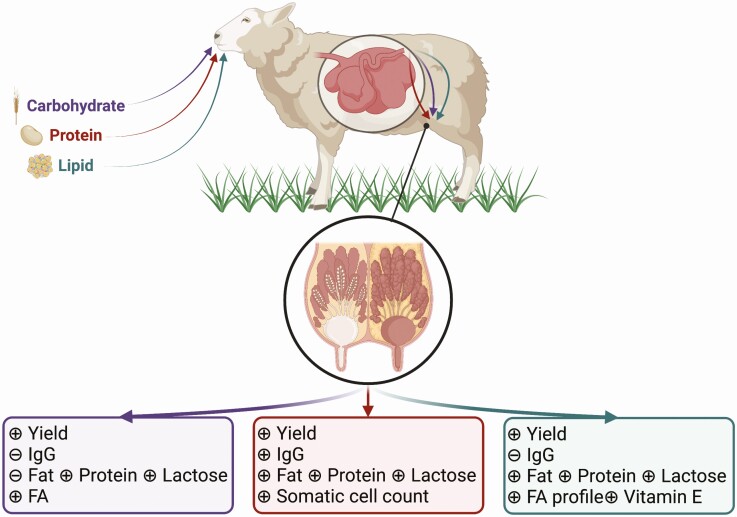
A schematic representation of how dietary carbohydrate (color-coded purple), protein (color-coded maroon), and fat (color-coded teal) intake affects colostrum yield and composition (immunoglobulin G [**IgG**], macronutrient, and bioactive components concentrations) produced by ewes. ⊝ Indicates that the dietary component does not affect that colostrum parameter whereas ⊕ indicates that the colostrum parameter is affected by that dietary component. The figure was created with Biorender.com.

Carbohydrates proportionally contribute the greatest amount of dietary energy in ruminant rations and, as such, relative starch and NDF substitutions have consequences for dietary energy intake that often confound colostrum production responses. Furthermore, studies that manipulate dietary starch and NDF content to achieve specific energy intakes differ in which energy partition they target (i.e., focusing on ME as compared to net energy [**NE**]). This is additionally complicated by using either empirical or mechanistic models to predict energy requirements and dietary sufficiency and which coefficients of energetic efficiency are used for the conversion of ME to NE for pregnancy or maintenance. Thus, utilization of either system can impose a bias on colostrum production responses between studies. Research evaluating carbohydrate consumption prior to parturition is shown in Table 1 and responses are shown in Figures 1 and 2.

**Table 1.** Summary of colostrum production responses to varying prepartum dietary carbohydrate strategies (starch source, supplementation and inclusion rate, starch–fibre substitutions, neutral detergent fibre [**NDF**], and forage source) across dairy, beef, and sheep models^a^

			Component concentration				Component yield				
Study^b^	Dietary strategy	Yield	IgG	Fat	CP	Lactose	IgG	Fat	CP	Lactose	Other
Dairy cattle											
1	Corn vs. wheat grain	—	↓	nd	↓	nd	—	—	—	—	—
2	Grass silage + concentrate vs. grass silage	nd	nd	nd	nd	nd	nd	—	—	—	—
3	2×2 factorial: concentrate vs. RP-CLA	—	nd	—	—	—	—	—	—	—	—
4	2 × 2 factorial: starch vs. stearic acid	nd	—	nd	nd	nd	—	—	—	—	—
5 and 6	Starch:NDF substitution	nd	↓	nd	nd	nd	nd	nd	nd	nd	↑insulin
7	High vs. low energy	—	nd	nd	nd	nd	—	—	—	—	↓IgA
8	High vs. low energy	nd	nd	nd	nd	nd	nd	nd	nd	nd	↓3′SL
9 and 10	Starch:NDF substitution	nd	↓	nd	—	—	—	↑ ^†^	—	—	↑insulin ↑↓FA ↓Vit B_12_
11	Starch:NDF substitution	nd	nd	—	—	—	—	—	—	—	—
12	2 × 2 factorial: energy vs. monensin	nd	nd	—	—	—	nd	—	—	—	—
13	2×2 factorial: energy vs. RSM	—	nd	—	—	—	—	—	—	—	—
Beef cattle											
14	ME titration	↑	↓	nd	↓	↑	↑	nd	nd	—	↑insulin
15	Dry-rolled corn supplementation	nd	—	nd	nd	nd	—	—	—	—	—
Ewes											
16	Cracked corn supplementation	↑	—	↓	↓	↑	—	—	↑	—	—
17	Starch vs. ME supplementation	↑	—	nd	nd	↑	—	—	—	—	—
18	Cracked corn vs. barley supplement	↑	—	nd	nd	↑	—	—	—	—	—
19	2 × 2 factorial: barley vs. temperament	nd	nd	nd	↓	↑	—	—	—	—	—
20	Energy/protein lick supplementation	↑	—	—	—	—	—	↑	↑	↑	—
21	High vs. low energy	—	nd	—	nd	—	—	—	—	—	—
22	High vs. low energy	↑	nd	nd	nd	—	nd	nd	nd	—	—
23	High vs. low energy	↑	—	nd	↓ ^†^	↑	—	—	—	—	—
24	100%ME vs. NE titration	nd	—	—	nd	—	—	—	—	—	↑↓FA

Notes: Arrows indicate the response relative to the treatment (first listed compared to second), where nd corresponds to no significant differences (*P* ≥ 0.05), † denotes a tendency (0.05 < *P* <0.10), and a dashed line indicates not reported.

^a^Abbreviations: immunoglobulin G (**IgG**), rumen-protected conjugated linoleic acid (**RP-CLA**), rapeseed meal (**RSM**), metabolizable energy (**ME**), net energy (**NE**), immunoglobulin A (**IgA**), 3′sialyllactose (**3**′**SL**), fatty acid (**FA**).

^b^Sources: 1. Fatahnia et al. (2012); 2. Dunn et al. (2017); 3. Eger et al. (2017) ; 4. Daneshvar et al. (2020); 5. Fischer-Tlustos et al. (2021b); 6. Fischer-Tlustos et al. (2022b); 7. Nowak et al. (2012); 8. Fischer-Tlustos et al. (2022a); 9. Duplessis et al. (2015); 10. Mann et al. (2016); 11. Richards et al. (2020); 12. Vasquez et al. (2021); 13. Tefsa et al. (1999); 14. Hare et al. (2022); 15. Tanner et al. (2023); 16. Banchero et al. (2004a); 17. Banchero et al. (2004b); 18. Banchero et al. (2007); 19. Hawken et al. (2012); 20. Olivera-Muzante et al. (2022); 21. Bonagurio Gallo et al. (2020); 22. Hashemi et al. (2008); 23. Banchero et al. (2006);S24. Campion et al. (2016) .

#### Colostrum yield.

Research in dairy cattle demonstrates that increasing dietary energy by shifting the starch:NDF ratio does not impact colostrum yield. Daneshvar et al. (2020) found that altering close-up diet starch inclusion from 20.8% to 25.8% by partially replacing wheat bran with corn and barley did not affect colostrum yield. Larger shifts in dietary starch content (magnitude of difference: approximately 12% to 15%DM) yielded similar colostrum production (Richards et al., 2020; Fischer-Tlustos et al., 2021b: milked within 12 h of calving) despite crude protein (**CP**) and MP intake not always being balanced across treatments (Richards et al., 2020). Although bovine colostrum yield is largely unaffected by late-gestation dietary protein intake (described below), it is unclear whether interactions occur between dietary energy and protein intake.

In contrast to dairy cattle, increasing dietary starch inclusion linearly from 3% to 27%DM (consequently providing between 92% and 118% predicted ME requirements, respectively) increased colostrum yield by 80% in Simmental-Angus crossbred beef cattle (Hare et al., 2021). These cattle were unfamiliar with milking, but colostrum was successfully milked completely within 30-60 min after calving following treatment with oxytocin (2 mL; Hare et al., 2021). While it is unclear why beef cattle colostrum production is more responsive to starch intake than dairy cattle, there are several speculative explanations. First, it must be recognized that the beef cattle are not dairy cattle: there are inherent physiological differences from selection pressure for milk (dairy) compared to peripheral tissue accretion (beef) that affect glucose partitioning between breed function. Speculatively, antepartum glucose portioning to the mammary gland may already be maximized in dairy cattle during the phase of colostrum lactose synthesis, such that the system is saturated prior to the addition of more glucose as a substrate, whereas the counter response might occur with beef breeds. Second, the differences between studies regarding DMI, starch source, and site of starch degradation would speculatively have a substantial role in influencing colostrogenesis.

In accordance with beef cattle, increasing dietary starch and energy intake in sheep consistently increased colostrum yield (Banchero et al., 2015). Banchero et al. (2004a, 2004b) strategically used cracked corn as a postruminally degradable starch source, demonstrating increased first-milking half-udder colostrum yield (at parturition) relative to unsupplemented ewes. All ewes were hand-milked immediately after lambing and the milked half was covered so that it was not available for nursing by the lamb. Notably, ewes supplemented with cracked or whole lupins produced a similar mass of colostrum as the control, despite their energy intake exceeding that of the control by 0.76 to 0.93 Mcal ME/d (Banchero et al., 2004b). These data indicate that starch intake has a more profound impact than ME consumption on colostrum production and, possibly, that the site of starch degradation (ruminal vs. postruminal) is important. Postruminally degradable starch sources are shown to increase peripheral glucose concentrations (Banchero et al., 2004a, 2004b; Hare et al., 2022), thereby increasing available substrate for lactose synthesis and osmotic draw of water into colostrum. Increased colostrum lactose concentration has been observed concurrently with greater colostrum yield (Banchero et al., 2004a, 2004b; Hare et al., 2021), along with corresponding elevations in plasma glucose (Banchero et al., 2004a; Hare et al., 2022). Albeit colostrum lactose concentration is relatively less than whole milk which may challenge the concept that it is a primary osmotic driver for colostrum yield; however, colostrum production is also notably less than milk production. That said, there are plausibly other substrates in colostrum that contribute to osmotic pressure, such as urea or albumin as examples.

#### Immunoglobulin G concentration and yield.

In dairy cattle, prepartum starch source has been shown to alter IgG_1_ and total IgG concentrations in colostrum (Fatahnia et al., 2012); thus, this factor should be considered when comparing colostral IgG concentrations between studies using different dietary starch sources. Nonetheless, IgG concentration in first-milking colostrum collected within 1 to 12 h after calving has been reduced by greater dietary starch inclusion or ME intake precalving (Mann et al., 2016; Fischer-Tlustos et al., 2021b; Hare et al., 2021). However, these differences in IgG concentration are not consistent across studies (Richards et al., 2020). This may be partly attributed to IgG measurement technique as some use radial immunodiffusion (Mann et al., 2016; Fischer-Tlustos et al., 2021b; Hare et al., 2021), whereas others estimate IgG using a colostrometer (Richards et al., 2020). It may be possible that inconsistent responses to starch intake could reflect differing starch sources (Fathania et al., 2012). Alternatively, variation might be attributed to the supplement feeding duration (Mann et al., 2016; Richards et al., 2020) relative to lactogenesis phases 1 and 2. Interestingly, differences in prepartum serum IgG concentrations from –5 to –1 wk prior to calving have been observed in response to concentrate supplementation (Dunn et al., 2017). Yet, there is still a lack of effect on first-milking colostrum IgG concentration or yield (Dunn et al., 2017), suggesting that serum IgG concentrations were sufficient in both groups to saturate the mammary receptor responsible for IgG transfer.

In ewes, studies have consistently demonstrated that increasing starch and energy decreases the concentration of colostrum CP (Banchero et al., 2015). However, it is unlikely that the observed differences in CP are due to changes in IgG concentration, given that the aforementioned studies have found no effect of starch supplementation on colostrum IgG.

#### Macronutrient concentrations and yields.

Hare et al. (2021) demonstrated that increasing the proportion of dietary starch fed to beef cattle affected colostrum fat concentration, decreased protein concentration, and increased colostrum lactose concentration. Yet, in dairy cattle, colostrum macronutrient composition (CP, fat, and lactose) is largely unaffected by starch inclusion (Dunn et al., 2017; Daneshvar et al., 2020; Fischer-Tlustos et al., 2021b). There is generally a lack of response on colostrum fat concentration and yield, although Mann et al. (2016) observed that fat yield tended to increase with greater ME intake. Furthermore, differences have been observed in fatty acid concentrations (discussed below).

In contrast to dairy cattle, the inclusion of cracked corn decreased ewe colostrum fat concentration in one study (Banchero et al., 2004a), and increasing starch and energy appears to consistently reduce CP and increase lactose concentrations in ewe colostrum (Banchero et al., 2015). Studies in ewes traditionally evaluate macronutrient concentrations but not yields. However, Olivera-Muzante et al. (2022) demonstrated that the provision of an energy/protein lick in late-gestation ewes increased yields of fat, CP, and lactose in first-milking colostrum collected at lambing following oxytocin administration. This suggests that as lactose concentrations increase, so does yield, consequently diluting other components. Increased colostrum lactose concentration is also associated with greater colostrum yield and reduced protein and Brix% in dairy cattle (Soufleri et al., 2021), supporting the concept that colostrum yield may dilute component concentrations. Future studies in both ewes and cattle should aim to evaluate both colostrum macronutrient concentrations and yields to determine total mammary gland output.

#### Bioactive components and micronutrients.

The impact of overfeeding energy on colostrum bioactive components has been well-characterized in dairy cattle. Feeding a ration targeting 100% predicted ME requirements prior to calving promoted the incorporation of preformed fatty acids (**FA**) while suppressing the synthesis of de novo FA relative to cows fed a ration that targets 125% or 150% predicted ME requirements prior to calving (Mann et al., 2016). These results demonstrate that negative energy balance prior to calving can induce the release of FA from body reserves for uptake by the mammary gland, resulting in a substitution in FA source, while total fat concentration is unchanged. Speculatively, the colostrum FA profile might have implications for neonatal calf development. Colostrum insulin concentration has also been shown to increase by approximately 20% to 30% (Mann et al., 2016; Fischer-Tlustos et al., 2021b) in response to increased starch provision and energy, respectively. This is not unexpected, given that energy intake affects plasma insulin concentrations, and elevated plasma insulin at 1 to 3 d prior to calving is positively correlated with colostrum insulin (Mann et al., 2016). Furthermore, a companion study to Mann et al. (2016) demonstrated that feeding at a target of 125% ME during close-up decreased colostrum vitamin B12 concentrations by 25.8% compared to feeding a target of 100% ME (Duplessis et al., 2015), which may occur due to shifts in ruminal bacterial populations in response to altered NDF and starch inclusion.

Increasing dietary starch inclusion has been shown to have no effect on colostrum total sialic acid concentrations or yields (Fischer-Tlustos et al., 2022b) when colostrum was completely milked within 10 h of calving; however, increasing dietary energy content caused a 16% reduction in total sialylated oligosaccharide concentrations (Fischer-Tlustos et al., 2022a). This suggests that increasing dietary starch does not alter the production of sialic acid for oligosaccharide synthesis. Instead, it may decrease the expression or activity of sialyltranferases that catalyzes the synthesis of acidic oligosaccharides in the bovine mammary gland.

### Prepartum dietary protein intake

Models that evaluate the sufficiency of prepartum *N* intake relative to net protein supply have not traditionally considered its impact on colostrum production. True protein requirements for colostrum production (based on net *N* output) are substantial but transient and, as such, presumed negligible relative to pregnancy and maintenance requirements. Regardless, numerous studies (shown in Table 2) have evaluated various strategies for prepartum dietary protein intake relative to colostrum production for dairy and beef cattle and ewes relative to colostrum production (Figures 1 and 2). We need to consider these results respective to the context of ruminant *N* metabolism and *N* source (for example, CP rather than ruminally degradable protein).

**Table 2.** Summary of colostrum production responses to varying prepartum dietary protein strategies (crude protein [**CP**], rumen-undegradable protein [**RUP**], and metabolizable protein [**MP**] content, protein source, and amino acid supplementation) across dairy, beef, and sheep models^a^

			Component concentration				Component yield				
Study^b^	Dietary strategy	Yield	IgG	Fat	CP	Lactose	IgG	Fat	CP	Lactose	Other
**Dairy cattle**											
1	Adequate vs. restricted protein supply	nd	nd	—	—	—	—	—	—	—	—
2	2 × 3 factorial: RSM vs. energy	—	nd	—	—	—	—	—	—	—	—
3	Moderate vs. low CP	—	nd	—	—	—	—	—	—	—	—
4	High vs. low sunflower meal inclusion	↑	—	—	—	—	—	—	—	—	—
5	Moderate vs. low CP	—	nd	nd	—	nd	—	—	—	—	—
6	MP titration	nd	—	—	—	—	—	—	—	—	—
7	2×2 factorial: protein vs. fat content	—	—	—	nd	—	—	—	—	—	—
8	Moderate vs. low CP	nd	—	—	—	—	—	—	—	—	—
9	RP-Lys supplementation	nd	nd	nd	nd	—	—	—	—	—	—
10	2 × 2 factorial: MP content vs. DMI	nd	nd	nd	nd	nd	—	nd	nd	nd	—
Beef cattle											
11	Adequate vs. restricted protein supply	—	nd	—	—	—	—	—	—	—	—
12	CP titration	—	nd	—	—	—	—	—	—	—	—
13	2×2 factorial: protein vs. energy	—	nd	—	—	—	—	—	—	—	—
14	Dietary protein [and energy] restriction	—	nd	—	—	—	—	—	—	—	—
15	Corn DDGS supplement	nd	—	—	—	—	—	—	—	—	—
16	CP supplement intake	—	nd	—	—	—	—	—	—	—	—
17,18	Excess vs. adequate MP	—	nd	↓	nd	nd	—	—	—	—	↑insulin
19	MP titration and RP-Met supplementation	—	nd	nd	nd	—	—	—	—	—	—
20	Urea supplementation	nd	—	nd	nd	nd	—	nd	nd	nd	—
Ewes											
21	RUP titration vs. protein source	nd	nd	—	nd	—	nd	—	nd	—	—
22	2 × 2 factorial: CP vs. RUP [source]^6^	↑	—	—	—	—	—	—	—	—	—
23	CP content vs. zinc-chelated methionine^7^	—	nd	nd	nd	↑^†^	—	—	—	—	↓SCC
24	CP supplementation	↑	nd	—	—	—	↑	—	—	—	—
25	CP source andRUP	nd	—	—	—	—	—	—	—	—	—
26	Excess vs. adequate CP	↓	—	—	—	—	—	—	—	—	—
27	2×2 factorial: CP vs. trimester^8^	nd	nd	nd	nd	nd	nd	nd	nd	nd	—
28	2 × 2 factorial: RUP vs. oil^9^	nd	↑ ^†^	—	nd	—	nd	—	nd	—	—
29	RUP titration	↑	—	↑	↑	—	—	↑	↑	—	—
30	High or low RUP(+other)	—	nd	—	—	—	—	—	—	—	—
31	RUP titration	↑	↑	nd	↑	↓	—	—	—	—	—
32	High vs. low MP content	nd	—	nd	nd	—	—	nd	nd	—	—
33	Starch vs. starch+protein	nd	nd	nd	nd	nd	nd	nd	nd	↑	—
34	2 × 3 factorial: RUP vs. flaxseed	nd	nd	—	—	—	—	—	—	—	—
35	Excess vs. adequate MP	↑	—	nd	↑	nd	—	↑	↑	↑	—

Notes: Arrows indicate the response relative to treatment (first listed compared to second), where nd corresponds to no significant differences (*P* ≥ 0.05) † denotes a tendency (0.05 < *P* < 0.10), and a dashed line indicates not reported.

^a^Abbreviations: immunoglobulin G (**IgG**), rapeseed meal (**RSM**), rumen-protected (**RP**), dry matter intake (**DMI**), dried distillers’ grains (**DDGS**), and somatic cell count (**SCC**).

^b^Sources: 1. Burton et al. (1984); 2. Tesfa et al. (1999); 3. Santos et al. (2001); 4. Mansooryar et al. (2011); 5. Toghyani and Moharrey (2015) ; 6. Amirabadi Farahni et al. (2017); 7. Jaurena and Moorby (2017); 8. Amirabadi Farahni et al. (2019); 9. Fehlberg et al. (2020) ; 10. Akhtar et al. (2022); 11. DeLong et al. (1979); 12. Blecha et al. (1981); 13. Olson et al. (1981) ; 14. Hough et al. (1990); 15. Kennedy et al. (2019); 16. Dafoe et al. (2020); 17. Hare et al. (2019); 18. Hare et al. (2022); 19. Lièvre (2020) ; 20. Prezotto and Thorson (2022) ; 21. Dawson et al. (1999); 22. Hall et al. (1992); 23. Hatfield et al. (1995); 24. O’Doherty and Crosby (1996); 25. Holst et al. (2005); 26. Ocak et al. (2005) ; 27. Wallace et al. (2006) ; 28. Annett et al. (2008); 29. Amanlou et al. (2010); 30. Redden et al. (2010); 31. Rezai et al. (2012) ; 32. Mousavi et al. (2016); 33. Kopp et al. (2020) ; 34. Ababraki et al. (2021); 35. Moradi et al. (2021)

#### Colostrum yield.

Apart from substituting sunflower meal for cottonseed meal as a protein source wherein the authors observed that greater sunflower meal increases first-milking colostrum yield (*iso*-nitrogenous and *iso*-energetic rations; Mansooryar et al., 2011), most data indicate that colostrum yield (0–1 h postcalving, full udder [Amirabadi Farahni et al., 2019] and right rear quarter [Kennedy et al., 2019]) does not differ due to late-gestation dietary protein intake in dairy and beef cattle (Amirabadi Farahni et al., 2019; Kennedy et al., 2019); although studies are limited for beef cattle and further validation is required. This might suggest that decreases in colostrum yield with global nutrient restriction (Logan, 1977; Petrie et al., 1984) are the consequence of lesser energy intake irrespective of protein intake. However, this argument is not straightforward since neither oversupply nor restriction of energy intake from either dietary starch or fat inclusion (discussed elsewhere) affects colostrum yield in cattle.

By contrast to cattle, colostrum yield in sheep appears more sensitive to protein supplementation during late gestation, with most studies indicating that increased protein intake (at times, in conjunction with greater RUP intake) increases colostrum yield (Hall et al., 1992; Amanlou et al., 2010). However, studies have also indicated that protein intake does not affect colostrum yield in ewes (Mousavi et al., 2016). Sheep models vary more with respect to breed (and, therefore, genetic differences in colostrum production) and fecundity (Hall et al., 1992), which could explain the above-described variation in response and sensitivity to dietary protein content and source. However, colostrum collection protocol may also confound responses in these studies through using the weigh-suckle-weigh method (which represents offspring appetite rather than colostrum yield) and high variation and lag (within 24 h postlambing) in time to measurement (Amanlou et al., 2010; Mousavi et al., 2016).

#### Immunoglobulin G concentration and yield.

As with colostrum yield, colostrum IgG concentration, as measured by radial immunodiffusion (Santos et al., 2001; Hare et al., 2019) or estimated using Brix (Akhtar et al., 2022), is unaffected by protein intake in dairy and beef cattle (Santos et al., 2001; Hare et al., 2019; Akhtar et al., 2022). Similarly, with ewes, studies have not found a connection between protein intake and colostrum IgG concentration (as measured by radial immunodiffusion; O’Doherty and Crosby, 1996), though there may be an association with consumption of fish oil relative to RUP intake where IgG was quantified by the zinc sulfate turbidity analysis (Annett et al., 2008). O’Doherty and Crosby (1996) found that IgG yield increased with CP supplementation because colostrum yield was greater. Future studies with beef or dairy cattle should evaluate colostrum IgG yield with protein intake, as no data are currently available. But currently, it appears that IgG concentration in colostrum is not responsive to late-gestation protein intake in ruminants.

#### Macronutrient concentrations and yields.

There are limited data regarding how prepartum protein intake affects colostrum macronutrient composition and yield in cattle. That said, from what is available, colostral fat, protein, and lactose concentrations are unaffected (Lièvre, 2020; Akhtar et al., 2022), nor are their yields (Akhtar et al., 2022). To note, Hare et al. (2019) fed isocaloric rations that differed only in MP content (133% vs. 100% predicted MP requirements) and found that colostrum fat concentration (hand-stripped within an hour of calving prior to nursing) was drastically reduced for beef heifers overfed MP relative to a control fed at requirements (3.4% vs. 7.0%, respectively). It is unclear why this response occurred, and is contradicted by Lièvre (2020), where primi- and multiparous beef cattle consumed titrated MP rations that marginally undersupplied or oversupplied MP relative to requirements (90%, 100%, or 110%MP requirements) when ME intake was consistent across treatments. To note, samples from these cattle were taken with greater variation in time to colostrum milking (1–8 h) and nursing status.

Comparatively, colostrum protein concentration has been reported to increase with protein supplementation in some studies with ewes (Amanlou et al., 2010), but not all (Annett et al., 2008; Mousavi et al., 2016). Results are similarly mixed with respect to colostrum fat and lactose concentrations (Amanlou et al., 2010; Mousavi et al., 2016). Again, unless colostrum yield is increased, macronutrient yield is unaffected by late-gestation protein intake (Mousavi et al., 2016).

#### Bioactive components and micronutrients.

Hare et al. (2022) observed that MP overconsumption increased colostrum insulin concentration, similar to what was observed when overfeeding ME (Mann et al., 2016; Fischer-Tlustos et al., 2021b). Work by Radford et al. (2018) exploring the colostrum proteome from cows oversupplied MP relative to the serum proteome of their calves was conducted in conjunction with Hare et al. (2019, 2022), in which colostrum samples were collected within an hour after calving before the calf had nursed. Serum was collected from calves prior to and 6-h after colostrum consumption. Cows that had been oversupplied MP prior to calving demonstrated shifts in the colostrum proteome to enhanced proteins associated with intestinal and immune system development and depletion of proteins associated with growth regulation. Furthermore, at 6-h after birth, 60 proteins were common between the calf sera proteome and the colostrum proteome, indicating possible absorption of additional colostrum proteins that exhibit modulation by prepartum MP intake. From calves born to MP-supplemented dams, there was an enhancement in targeted responses to antigens to which their dam is immuno-reactive, and less proteins associated with a nonspecific inflammatory response. Shifts in antepartum metabolism from prepartum nutrient intake appear to have the capacity to alter the colostral bioactive profile even when gross changes in colostrum yield and composition are not present; thus, prepartum protein intake should not be disregarded as there are likely downstream impacts on the neonatal calf.

### Prepartum dietary fat intake

Rations typically have 2%DM dietary fat and, generally, dietary fat should not exceed 6% to 7%DM to avoid detrimental impacts on ruminal fermentation. However, greater dietary fat provision can also improve ration palatability, control dust and fines, and alleviate heat stress due to its lesser heat increment respective to proteins and carbohydrates. Increasing prepartum dietary fat intake can concurrently increase energy intake without providing excessive dietary starch. Consequently, prepartum fat intake may have utility for prepartum rations and studies in cattle (Table 3) and ewes have described various colostrogenic responses (Figures 1 and 2) to prepartum dietary fat inclusion and source.

**Table 3.** Summary of colostrum production responses to varying dietary fat inclusion strategies (source, fatty acid [**FA**] profile, and inclusion rate) across dairy, beef, and sheep models^a^

			Component concentration				Component yield				
Study^b^	Dietary strategy	Yield	IgG	Fat	Protein	Lactose	IgG	Fat	Protein	Lactose	Others
Dairy cattle											
1	Fat source comparison	—	—	—	—	—	—	—	—	—	↑ antigen
2	Linseed oil vs. control	—	—	—	—	—	—	—	—	—	↑↓FA
3	FA type vs. control	—	↑	—	—	—	—	—	—	—	↑↓FA
4	Fat source vs. control	—	↑	↓	↑ ^†^	↓ ^†^	—	—	—	—	↑↓FA
5	2×2 factorial: concentrate vs. RP-CLA	—	nd	—	—	—	—	—	—	—	—
6	2 × 2 factorial: protein vs. fat	—	nd	—	nd	—	—	—	—	—	—
7	Fat source vs. control	nd	↑	nd	nd	nd	—	—	—	—	↑↓FA
8	2 × 2 factorial: starch vs. stearic acid	nd	—	↓ ^†^	nd	nd	—	—	—	—	—
9,10, and 11	Fat source/inclusion rate vs. control	—	nd	↓	↑	nd	—	—	—	—	↑↓FA
12	Fat source vs. control	nd	↓	—	nd	—	nd	—	—	—	nd
Beef cattle											
13	Fat source vs. control	—	nd	nd	nd	nd	—	—	—	—	—
14	EPA&DHA vs. control	—	—	—	—	—	—	—	—	—	↑↓FA
15	RP-SBO vs. control	—	↑	—	—	—	—	—	—	—	↑↓FA
16	Bypass EFA vs. control	—	↑	—	—	—	—	—	—	—	—
Ewes											
17	PUFA vs. control	—	—	—	—	—	—	—	—	—	↑↓FA
18 and 19	2 × 2 factorial: fat source vs. vit E	↓	—	↓	nd^†^	—	—	↓	↓	—	↑↓FA, ↑Vit E
20	Fish oil inclusion vs. RUP	↓	nd	—	nd	—	↓	—	↓	—	↑↓FA
21	2 × 2 factorial: fat source vs. concentration	nd	nd	nd	nd	nd	nd	nd	nd	nd	↑↓FA
22	Fish meal vs. control	—	—	nd	nd	—	—	—	—	—	↑↓FA
23	DHA vs. control	—	↑	↓	nd	↑	—	—	—	—	—
24	SBO titration	↑	—	nd	nd	nd	—	—	—	—	—
25	RP-EPA&DHA vs. RP-palm	—	—	—	—	—	—	—	—	—	↑↓FA
26	Fat source/form vs. control	↑^†^	—	nd	nd	↓	—	↑	↑	nd	—
27	Excess energy [source] vs. adequate control	↑	—	nd	nd	nd	—	—	—	—	—
28	ALA vs. control	—	nd	↓^†^	nd	nd	—	—	—	—	↑↓FA
29	FA source vs. control	—	—	nd	nd	nd	—	—	—	—	—

Notes: Arrows indicate the response relative to treatment (first listed compared to second), where nd corresponds to no significant differences (*P* ≥ 0.05) † denotes a tendency (0.05 < *P* < 0.10), and a dashed line indicates not reported.

^a^Abbreviations: immunoglobulin G (**IgG**), rumen-protected (**RP**), conjugated linoleic acid (**CLA**), eicosapentaenoic acid (**EPA**), docosahexaenoic acid (**DHA**), soybean oil (**SBO**), essential fatty acid (**EFA**), polyunsaturated fatty acid (**PUFA**), and ɑ-linolenic acid (**ALA**).

^b^Sources: 1. Lessard et al. (2004); 2. Santschi et al. (2009); 3. Garcia et al. (2014); 4. Salehi et al. (2016); 5. Eger et al. (2017); 6. Jaurena and Moorby (2017); 7. Jolazadeh et al. (2019); 8. Daneshvar et al. (2020); 9. Vogel et al. (2020) ; 10. Liermann et al. (2020); 11. Uken et al. (2021) ; 12. Sun et al. (2022); 13. Dietz et al. (2003) ; 14. Mašek et al. (2014); 15. Brandão et al. (2020); 16. Ricks et al. (2020); 17. Noble et al. (1978); 18. Capper et al. (2005); 19. Capper et al. (2006); 20. Annett et al. (2008); 21. Annett et al. (2009); 22. Or-Rashid et al. (2010); 23. Keithly et al. (2011); 24. Marcías-Cruz et al. (2017); 25. Coleman et al. (2018); 26. Ababakri et al. (2021); 27. Moradi et al. (2021); 28. Averós et al. (2022); 29. Nurlatifah et al. (2022).

#### Colostrum yield.

Late-gestation fat inclusion rate and source have not been shown to affect first-milking colostrum production in dairy cattle, either when NE concentration was maintained (Sun et al., 2022) or when it was not (Daneshvar et al., 2020). To the authors’ knowledge, colostrum yield in beef cattle fed differing fat inclusion rates or sources has not been reported.

First-milking colostrum yield in ewes appears to be more responsive to dietary fat content than cattle, but results across studies remain inconsistent. Some authors have found that specific fat sources (fish oil relative to Ca-salts of palmitic acid) can decrease first-milking half-udder colostrum yield collected within an hour (Annett et al., 2008) and between 12 and 16 h after lambing (Capper et al., 2006). Conversely, others have found titrated increases in soybean oil inclusion (Marcías-Cruz et al. (2017) and extruded relative to whole flaxseed (Ababakri et al., 2021) cause first-milking (at parturition) colostrum yield to increase. Thus, it tentatively appears that elevated soybean or flaxseed inclusion (containing relatively higher concentrations of linoleic and *ɑ*-linolenic) in late-gestation rations could be beneficial for increasing ewe colostrum yield. Currently, it is unclear why this response occurs.

#### Immunoglobulin G concentration and yield.

Interestingly, colostral IgG concentration, as measured by ELISA or estimated using Brix, appears to be responsive to dietary fat inclusion and source in both dairy (Salehi et al., 2016) and beef cattle (Brandão et al., 2020; Ricks et al., 2020). Most authors report elevated (Salehi et al., 2016; Brandão et al., 2020; Ricks et al., 2020) rather than suppressed colostrum IgG concentrations (quantified with an ELISA; Sun et al., 2022). Elevated colostrum IgG concentration from prepartum fat intake is consistent among studies that feed supplements with elevated quantities of linoleic acid and uniformly increased plasma linoleic acid concentrations in supplemented cattle (Brandão et al., 2020; Ricks et al., 2020). Colostrum IgG yield is often not reported in dairy and beef cattle, although one study noted that there was no difference in colostrum IgG output between treatments, despite colostrum IgG concentration being decreased (Sun et al., 2022).

Although fewer data are available for ewes, their colostral IgG concentration does not appear to respond to fat intake and source as in cattle (Annett et al., 2008). Though, as implied above, this may be due to the inclusion of fat sources that have relatively lower polyunsaturated fatty acid concentrations. Immunoglobulin G yield has only been reported to decrease when colostrum yield was reduced by fish oil inclusion (Annett et al., 2008).

#### Macronutrient concentrations and yields.

Colostral macronutrient concentrations are usually unaltered by dietary fat content and source for cattle (Jaurena and Moorby, 2017; Daneshvar et al., 2020). Notably, when the macronutrient profile was affected, colostrum fat concentration was consistently reduced by increased dietary fat provision and source (Salehi et al., 2016; Daneshvar et al., 2020). Dietary fat intake and source also increased colostrum protein concentration (Salehi et al., 2016). Salehi et al. (2016) also reported that supplemental canola, rather than sunflower seed, tended to increase colostrum lactose concentration. We are unaware of any studies in dairy or beef cattle that describe macronutrient yields relative to prepartum fat source and intake. Ewe colostrum macronutrient composition appears to be similarly unaffected by late-gestation dietary fat intake (Marcías-Cruz et al., 2017) With some exception (Capper et al., 2006).

#### Bioactive components and micronutrients.

The colostral FA profile is the most reported bioactive component among research evaluating prepartum dietary fat intake and source. For both cattle and sheep, colostrum FA composition ubiquitously varies across all reported studies when the dietary fat inclusion rate or source is altered in such a way that coincides with dietary FA intake and maternal plasma FA profile. With respect to other bioactive components and micronutrients, Capper et al. (2006) observed that colostrum vitamin E concentration was affected by the interaction of fat source with vitamin E supplementation, such that ewes consuming Ca-salts of palmitic acid supplemented with vitamin E had substantially larger concentrations (approximately 3- to 4-fold increase) and outputs (approximately 2.7- to 3.1-fold greater) of vitamin E in their colostrum. As reported by Sun et al. (2022), feeding dairy cattle iso-energetic and -nitrogenous diets containing extruded flaxseed, soybean, or lacking in both, had no effect on the concentrations of lactoferrin, lysozyme, peroxidase, Zn, or Ca in colostrum. As with prepartum protein intake, more research is required to understand shifts in the bioactive components available to neonatal ruminants when gross colostrum composition is unaltered.

## Conclusion

Prepartum nutrient intake has the capacity to influence colostrogenesis in bovine and ovine species and impact colostrum yield and composition. Although ewes are generally more sensitive to ration composition than cattle, there is evidence to suggest that prepartum nutrition can be used strategically to promote colostrum production in both species. Dietary starch and fat content and source appear to exert influence on colostrum IgG concentration, with data indicating that starch:NDF substitutions affects colostrum yield in beef but not dairy cattle. Current data suggest that cattle are irresponsive to dietary protein content, whereas ewe colostrum production responds to dietary protein source and intake. Further work needs to differentiate between carbohydrate, protein, and fat sources, accounting for total IgG and macronutrient yield, to characterize mammogenic capacity under differing nutritional regimens, particularly in relation to the feeding duration and timing of supplementation relative to the onset of colostrogenesis. In addition, there is a need to detail the colostral bioactive profiles, as this constituent often responds to prepartum nutrient intake without changes in the gross composition of colostrum. Colostral bioactive components are inherently important for neonatal development and, as such, prepartum nutrient intake might be used strategically to benefit the offspring.

## About the Authors

**Koryn Hare** comes from a rural community in southwestern Saskatchewan where she has a cow-calf herd and completed her BSc Agriculture (2015) and MSc Animal Science (2018) at the University of Saskatchewan. She joined Dr. Steele’s lab and began her Ph.D. at the University of Guelph in 2018. Her research interests revolve around how prepartum nutrition affects colostrum production in cattle and the influence of colostrum bioactive components on neonatal calf development.

**Amanda Fischer-Tlustos** completed her MSc Animal Science at the University of Alberta in 2017, focusing on the effects of certain colostrum management practices on the physiology of the newborn dairy calf. After working in various roles, including as a research assistant, conference coordinator, and content writer, she began her PhD in May 2020 under the supervision of Drs. Michael Steele and John Cant. Her PhD research focuses on maternal factors controlling the synthesis and concentrations of bioactive compounds in bovine colostrum and transition milk and their role in improving dairy calf immunity and development.

**Dr. Katie Wood** developed a passion for animal agriculture early in life through her experiences on her family’s cow/calf operation and through her involvement in the 4-H program. After concluding her PhD at the University of Guelph, she completed an NSERC postdoctoral fellowship at the University of Saskatchewan. During this time her work focused on understanding gut barrier function and nutrient absorption in ruminants and their interactions with health, metabolic disease and production efficiencies. Dr. Wood returned to the University of Guelph in April 2016 as an Assistant Professor, with her research interests centered around physiological process that influence feed efficiency and production in feedlot cattle, as well as how late-gestational nutrition can impact cow health and production.

**Dr. John Cant** John grew up in the 1970s on an 11-acre, self-sufficient farm outside Sackville, New Brunswick. Dr. Cant completed his PhD at University of California Davis under the supervision of Drs. Ed DePeters and Lee Baldwin, where he studied a dietary fat-induced milk protein depression and learned mathematical modelling. In 1996, after a postdoctoral stint with Dr. Brian McBride at the University of Guelph, where he built a computer model to predict lactational performance of the dairy cow for Agribrands Purina, he was hired as a faculty member at The University of Guelph. His research has focused on the regulation of milk synthesis and the transformation of dietary nutrients into milk.

**Dr. Michael Steele** is a professor at the University of Guelph, with his research aiming to explain ruminant physiology through a systems approach from the molecular to whole animal level. His research program is inspired by his upbringing on a progressive dairy farm in Southwestern Ontario, where he became fascinated by the integration of advanced scientific technologies and livestock production systems. The overall objective of his research program is to develop a better understanding of how preweaning nutritional and management factors that are common in livestock production can impact gastrointestinal development and metabolism during the pre-weaning phase and later in life.
